# Spatiotemporal variations in length-weight relationship, growth pattern and condition factor of* Periophthalmus variabilis* Eggert, 1935 in Vietnamese Mekong Delta

**DOI:** 10.7717/peerj.12798

**Published:** 2022-01-11

**Authors:** Quang Minh Dinh, Ton Huu Duc Nguyen, Tien Thi Kieu Nguyen, Giang Van Tran, Ngon Trong Truong

**Affiliations:** 1 Department of Biology, School of Education, Can Tho University, Xuan Khanh Ward, Ninh Kieu District, Can Tho, Vietnam; 2 Department of Biology, An Khanh High School, Ninh Kieu District, Can Tho, Vietnam; 3 Department of Zoology, Faculty of Biology, Hue University of Education, Hue City, Vietnam; 4 Department of Molecular Biology, Biotechnology Research and Development Institute, Can Tho University, Xuan Khanh Ward, Ninh Kieu District, Can Tho, Vietnam

**Keywords:** Positive allometry, Isometry, Vietnam, Dusky-gilled mudskipper

## Abstract

Length-weight relationship (*LWR*), growth pattern and condition factor (*CF*) play a vital role in fish resource evaluation and management but data on this is limited for *Periophthalmus variabilis*. This is an amphibious fish that lives in the mudflats of the mangrove forests in the Western Pacific regions, including the Vietnamese Mekong Delta (VMD). This paper contributes to the understanding of the *LWR*, growth pattern and *CF* of *P. variabilis*. Fish specimens were collected by hand at four sites in the VMD from April 2020 to March 2021. The analysis of 495 individual fish (259 females and 236 males) showed that fish weight could be estimated from a given fish length due to high determination values (*r*^2^ = 0.70 − 0.97). Dusky-gilled mudskippers of the VMD exhibited positive allometry, as indicated by their larger than 3 *b* value (*b* = 3.094 ± 0.045, *p* = 0.04). However, the growth pattern of the mudskipper between the sex and maturation stage (immature versus mature) showed isometry. These fish displayed positive allometry in the dry season (*b* = 3.138 ± 0.065) (>3, *p* = 0.04) but isometry in the wet season (*b* = 3.058 ± 0.061) (≈3, *p* = 0.34). Fish growth ranged from isometry to positive allometry and varied by site (*b* = 2.850 ± 0.103–3.370 ± 0.114) and month (*b* = 2.668 ± 0.184–3.588 ± 0.299) based on the b value. The *CF* of *P. variabilis* was not affected by sex (*p* = 0.29), body size (*p* = 0.64) or season (*p* = 0.43), but was affected by site (*p* = 0.01) and month (*p* = 0.01). The *CF* of this species (1.05 ± 0.02) was higher than 1 (*p* < 0.001), indicating that the fish adapted well to their habitat.

## Introduction

The length-weight relationship (*LWR*) plays an essential role in both evaluating the growth and biomass of a fish population (Khaironizam & Norma-Rashid, 2002; Mahmood et al., 2012; Jin et al., 2015; Dinh, 2016a; Lam & Dinh, 2021) and in assessing fishery management (Froese, 1998; Froese & Pauly, 2000; Gonzalez Acosta, De La Cruz Agüero & De La Cruz Agüero, 2004; Jin et al., 2015; Phan et al., 2021). In addition, the growth pattern and the *CF* play a vital role in understanding the ecological adaptations of fish (Abdoli et al., 2009; Dinh et al., 2016). The growth pattern and the *CF* of some fish, including gobies, are affected by sexual, intraspecific and spatiotemporal variables (Froese, 2006; Abdoli et al., 2009; Lam & Dinh, 2021; Phan et al., 2021; Truong et al., 2021). However, this data is limited for mudskippers, one of the gobiid fish groups in the Vietnamese Mekong Delta (VMD).

The mudflats and mangroves are habitats for a number of animal species including fish (Sanders et al., 2010; Sasmito et al., 2020). The mudskipper is a unique fish group that lives mainly in these habitats (Murdy, 1989) and can obtain oxygen directly from the air using their skin and gills (Jaafar, Perrig & Ming Chou, 2009). *Periophthalmus* is one of the largest genera of mudskippers with 19 described species found throughout the world (Murdy & Jaafar, 2017). *Periophthalmus* of the VMD is comprised of three species: *P. chrysospilos*, *P. gracilis* and *P. variabilis* (Tran et al., 2013). Of these three species, *P. variabilis* is found quite frequently in mudflat and mangrove regions (Murdy, 1989; Kottelat et al., 1993; Jaafar & Hou, 2012; Tran et al., 2013; Tran et al., 2020; Tran & Dinh, 2021; Tran et al., 2021a) and can move flexibly in and out of the water to catch prey (Wicaksono et al., 2020). In the VMD, our observations show that the number of individual *P. variabilis* has decreased over time. However, there is no data on its biology and ecology. This study was conducted to document fish LWR, growth pattern and *CF* to further understand the ecological adaptations of the *P. variabilis* population.

## Materials and Methods

### Study site and fish analysis

**Figure 1:**
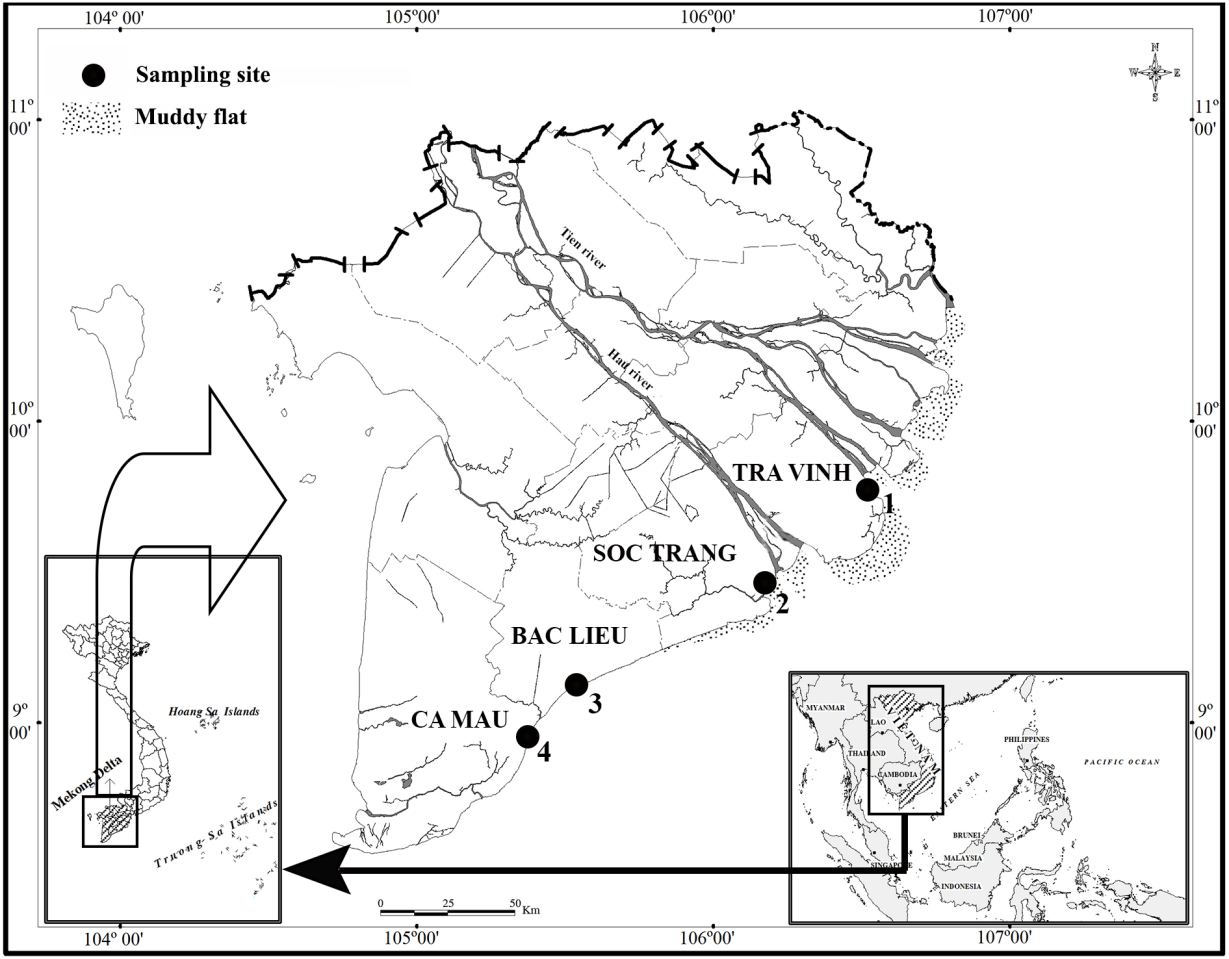
Distribution map of sampling points. •: Sampling site; 1: Duyen Hai–Tra Vinh; 2:Tran De–Soc Trang; 3: Dong Hai–Bac Lieu; 4: Dam Doi–Ca Mau.

The present research was carried out in four locations along the estuarine and coastal regions in the VMD, including Duyen Hai–Tra Vinh (DHTV, 9°40′29.5″N 106°34′49.5″E); Tran De–Soc Trang (TDST, 9°26′19.7″N 105°10′48.1″E); Dong Hai–Bac lieu (DHBL, 9°05′50.5″N 105°29′54.7″E) and Dam Doi–Ca Mau (DDCM, 8°58′10.4″N 105°22′58.9″E) (Fig. 1). There are two seasons at these sites: the dry season from January to May and the wet season from June to December (Le et al., 2006). The dominant plants in DHTV, TDST and DHBL are *Sonneratia caseolaris*, *Avicennia marina* and *Bruguiera gymnorrhiza*, respectively. In DDCM, *A. marna* and *B. gymnorrhiza* are equally dominant (Dinh et al., 2021a). The pH ranged from 7.6–8.0, and the salinity varied widely from 12.3 to 23.5%. The pH varied with the site but not with the season, whereas the salinity varied with the season but not the site (Dinh et al., 2021a).

Fish samples were collected monthly by hand for 4-hours at a time in an area of 120 m^2^ (6-m width × 20-m length) from April 2020 to March 2021. Fish specimens were easily distinguished from congeners using their external features (Murdy & Jaafar, 2017). *Periophthalmus variabilis* are covered by many irregular blackish dots, whereas *P. chrysospilos* and *P. gracilis* are covered by small orange spots and are greyish brown with several irregular narrow white bars, respectively. The roughly triangular first dorsal fin is found on *P. variabilis* and *P. chrysospilos*, but not on *P. gracilis*. Filamentous spines are found in the first dorsal fin of *P. chrysospilos* but are not found in *P. variabilis*. MS222 was used to anaesthetize fish specimens before being preserved in a 5% formalin buffer and shipped to the laboratory. In the laboratory, fish sex was differentiated using genital papilla, *e.g.*, a triangle shape in males and an oval shape in females. Fish total length (*TL*) was measured to the nearest 0.1 cm using a ruler, and fish weight (*W*) was recorded to the nearest 0.01 g using an electric scale.

### Data analysis

The *LWR* was determined using the formula *W* = *a* ×*TL*^*b*^ (*W*: fish weight, *a*: intercept parameter, *TL*: fish total length, and *b*: slope parameter) (Ricker, 1973). The condition factor (*CF*) was calculated as suggested by Le Cren (1951) using the formula *CF* = *W*/(*a* ×*TL*^*b*^).

The quality of *LWR* s was determined by using the determination coefficient (*r*^2^) (Metin et al., 2011). The *t*-test was performed to verify if the *b* value obtained from the *LWR* s was close to a cubic value. Species displayed positive allometry (*b* > 3), negative allometry (*b* < 3) and isometry (*b* =3) (Martin, 1949). The *t*-test was applied to confirm whether the *CF* varied by sex, size and season, while one-way ANOVA was used to assess any variations in th *CF* between months and sites (Mahmood et al., 2012). The *t*-test was used to verify if the *CF* was significantly different from the threshold of 1. A General Linear Model was used to confirm if the interactions of sex × season, sex × site and season × site affected the *CF* (Dinh, 2016a). The length at first maturity (*L*_*m*_) was used to divide fish into the immature group if *TL* < *L*_*m*_ and into the mature group if *TL* ≥ *L*_*m*_ (*e.g.*, *L*_*m*_ values of females and males were 5.0 cm and 5.7 cm in DHTV; 4.6 cm and 5.8 cm in TDST; 4.9 cm and 5.2 cm in DHBL; and 6.2 cm and 5.9 cm in DDCM, unpublished data). SPSS v.21 was used for data analysis and all tests were set at *p* < 0.05. The Benjamini–Hochberg procedure was used to decrease the Type I error of all tests (Benjamini & Hochberg, 1995; McDonald, 2014).

## Results

### Length-weight relationship and growth pattern

A total of 495 individuals (TL: 3.7–8.5 cm; W: 0.31–7.09 g) were collected from the four sites, as shown in Table 1. The fish weight of males and females in different sizes, seasons, months and sites could be estimated from fish length due to high determination values (*r*^2^ = 0.70–0.97, Table 2). The *LWR* of *P. variabilis* (female, male and both sexes) was presented in Table 2 as *W* = *aTL*^*b*^. As the slope parameters (*b*) obtained from the *LWR* of males (3.123 ± 0.068, *n* = 236) and females (3.014 ± 0.064, *n* =259) were not significantly different from the threshold of 3 (*df*_*male*_ = 231, *t*_*male*_ = 1.81, *p*_*male*_ = 0.07; *df*_*female*_ =257, *t*_*female*_ = 0.22, *p*_*female*_ = 0.83, Table 2), both males and females displayed an isometric growth pattern. Similarly, the growth pattern of the fish was not affected by its developmental stages since the immature and mature fish both exhibited isometry with *b* values (2.964 ± 0.071, *n* = 404) close to 3 (*df*_*immature*_ =89, *t*_*immature*_ = 1.40, *p*_*immature*_ = 0.17; *df*_*mature*_ = 402, *t*_*mature*_ = 0.51, *p*_*mature*_ = 0.61, Table 2). In contrast, the mudskipper displayed isometry in the wet season as *b* (3.058 ± 0.061, *n* = 283) was ≈3 (*df* = 281, *t* = 0.95, *p* = 0.34) but had positive allometry in the dry season since *b* (3.138 ± 0.065, *n* =212) was >3 (*df* = 210, *t* = 2.21, *p* = 0.03). In the northern region of the Hau river mouth, *P. variabilis* exhibited isometry since the *b value was* ≈3 (*b*_*DHTV*_ = 3.007 ± 0.061, *n*_*DHTV*_ = 194, *df*_*DHTV*_ = 192, *t*_*DHTV*_ = 0.12, *p*_*DHTV*_ = 0.91; *b*_*TDST*_ = 2.850 ± 0.103, *n*_*TDST*_ =119, *df*_*TDST*_ = 117, *t*_*TDST*_ = −1.46, *p*_*TDST*_ = 0.15, Table 2), but had positive allometry in the southern region because *b was >* 3 (*b*_*DHBL*_ = 3.370 ± 0.114, *n*_*DHBL*_ =89, *df*_*DHBL*_ = 87, *t*_*DHBL*_ = 3.25, *p*_*DHBL*_< 0.001; *b*_*DDCM*_ = 3.232 ± 0.101, *n*_*DDCM*_ = 93, *df*_*DDCM*_ = 91, *t*_*DDCM*_ = 2.30, *p*_*DDCM*_ = 0.02, Table 2). The species displayed isometry in April and again from August through December as the *b* value (2.668 ± 0.184 - 3.001 ± 0.105) was close to 3 (*t*-test, *p* > 0.05 for all cases, Table 3), but displayed positive allometry for the remaining months since the *b* value (3.215 ± 0.086–3.771 ± 0.325) was significantly higher than 3 (*p* < 0.05 for all cases, Table 3). Overall, the Dusky-gilled mudskipper displayed positive allometry as the *b* value (3.094 ± 0.045) was >3 (*n* = 495, *df* = 493, *t* = 2.09, *p* = 0.04).

**Table 1:** Number of samples by sex, site and month.

Months	Duyen Hai–Tra Vinh		Tran De–Soc Trang		Dong Hai–Bac Lieu		Dam Doi–Ca Mau	
	Male	Female	Male	Female	Male	Female	Male	Female
Apr-20	12	6	4	5	1	4	1	2
May-20	6	8	5	3	5	1	2	3
Jun-20	12	15	5	5	4	1	6	1
Jul-20	9	5	4	4	6	3	1	5
Aug-20	6	6	6	8	5	5	2	4
Sep-20	6	8	3	6	4	3	5	6
Oct-20	4	9	6	6	2	4	5	3
Nov-20	6	6	5	3	3	4	2	6
Dec-20	8	3	1	10	4	3	8	3
Jan-21	4	22	3	7	3	6	3	8
Feb-21	8	7	4	6	8	2	3	6
Mar-21	9	9	5	5	5	3	6	2
Total	90	104	51	68	50	39	44	49

**Table 2:** Variations of growth pattern of *P. variabilis* by sex, season, size and site (*TL*, total length; *W*, body weight; *n*, number of individuals, *b*, slope parameter; a, intercept parameter; *r*^2^, determination parameter; *df*, freedom degree).

Fish groups		*TL*	*W*	*n*	*b*± SE	*a*± SE	*r* ^2^	*t-value*	*df*	*p-value*	Growth type
Gender	Female	3.7–8.5	0.31–7.09	259	3.014 ± 0.064	0.010 ± 0.001	0.90	0.83	257	0.22	Isometry
	Male	4.0–7.9	0.44–4.86	236	3.123 ± 0.068	0.008 ± 0.001	0.90	0.07	234	1.81	Isometry
Season	Dry	3.7–8.5	0.31–7.09	212	3.138 ± 0.065	0.008 ± 0.001	0.92	0.03	210	2.12	Positive allometry
	Wet	3.8–8.3	0.41–6.79	283	3.058 ± 0.061	0.009 ± 0.001	0.90	0.34	281	0.95	Isometry
Fish size	Immatury	3.7–5.7	0.31–2.68	91	3.327 ± 0.234	0.006 ± 0.002	0.70	0.17	89	1.40	Isometry
	Matury	5.2–8.5	1.20–7.09	404	2.964 ± 0.071	0.011 ± 0.001	0.81	0.61	402	−0.51	Isometry
Study site	Duyen Hai, Tra Vinh	3.8–7.8	0.41–4.76	194	3.007 ± 0.061	0.010 ± 0.001	0.93	0.91	192	0.12	Isometry
	Tran De, Soc Trang	4.1–7.9	0.44–4.86	119	2.850 ± 0.103	0.013 ± 0.003	0.87	0.15	117	−1.46	Isometry
	Dong Hai, Bac Lieu	3.7–7.5	0.31–4.86	89	3.370 ± 0.114	0.005 ± 0.001	0.91	0.00	87	3.25	Positive allometry
	Dam Doi, Ca Mau	7.1–8.5	0.78–7.09	93	3.232 ± 0.101	0.006 ± 0.001	0.92	0.02	91	2.30	Positive allometry
Total		3.7–8.5	0.31–7.09	495	3.094 ± 0.045	0.008 ± 0.001	0.91	2.09	493	0.04	Positive allometry

**Table 3:** Variations of growth pattern of *P. variabilis* by months (*TL*, total length; *W*, body weight; *n*, number of individuals; *b*, slope parameter; a, intercept parameter; *r*^2^, determination parameter; *df*, freedom degree).

Months	*TL*	*W*	*n*	*b*± SE	*a*± SE	*r* ^2^	*t-value*	*p*	*df*	Growth type
Apr-20	5.6-7.3	1.00-4.66	35	3.588 ± 0.299	0.003 ± 0.002	0.81	2.57	0.01	33	Positive allometry
May-20	5.6-7.3	1.00-4.86	33	3.771 ± 0.325	0.002 ± 0.001	0.81	2.37	0.02	31	Positive allometry
Jun-20	3.9-7.7	0.44-4.76	49	3.390 ± 0.091	0.005 ± 0.001	0.97	4.29	0.00	47	Positive allometry
Jul-20	3.8-7.7	0.41-4.86	37	3.354 ± 0.143	0.005 ± 0.001	0.94	2.48	0.02	35	Positive allometry
Aug-20	4.8-8.3	1.30-6.89	42	2.887 ± 0.198	0.013 ± 0.005	0.84	−0.57	0.57	40	Isometry
Sep-20	4.5-7.8	1.02-4.40	41	2.767 ± 0.234	0.015 ± 0.006	0.78	−1.00	0.33	39	Isometry
Oct-20	4.3-7.8	0.82-4.71	39	2.668 ± 0.184	0.018 ± 0.006	0.85	−1.80	0.08	37	Isometry
Nov-20	4.7-8.2	1.22-6.79	35	2.772 ± 0.210	0.016 ± 0.006	0.84	−1.09	0.29	33	Isometry
Dec-20	4.10-7.7	0.62-4.41	40	3.001 ± 0.105	0.009 ± 0.002	0.96	0.01	0.99	38	Isometry
Jan-21	4.1-8.5	0.50-7.09	56	3.215 ± 0.086	0.006 ± 0.001	0.96	2.50	0.02	54	Positive allometry
Feb-21	3.7-7.9	0.31-4.61	44	3.418 ± 0.086	0.004 ± 0.001	0.97	4.86	0.00	42	Positive allometry
Mar-21	3.9-7.3	0.57-4.38	44	3.229 ± 0.090	0.008 ± 0.001	0.97	2.54	0.01	42	Positive allometry
Total	3.7-8.5	0.31-7.09	495	3.094 ± 0.045	0.008 ± 0.001	0.91	2.09	0.04	493	Positive allometry

### Condition factor

**Figure 2:**
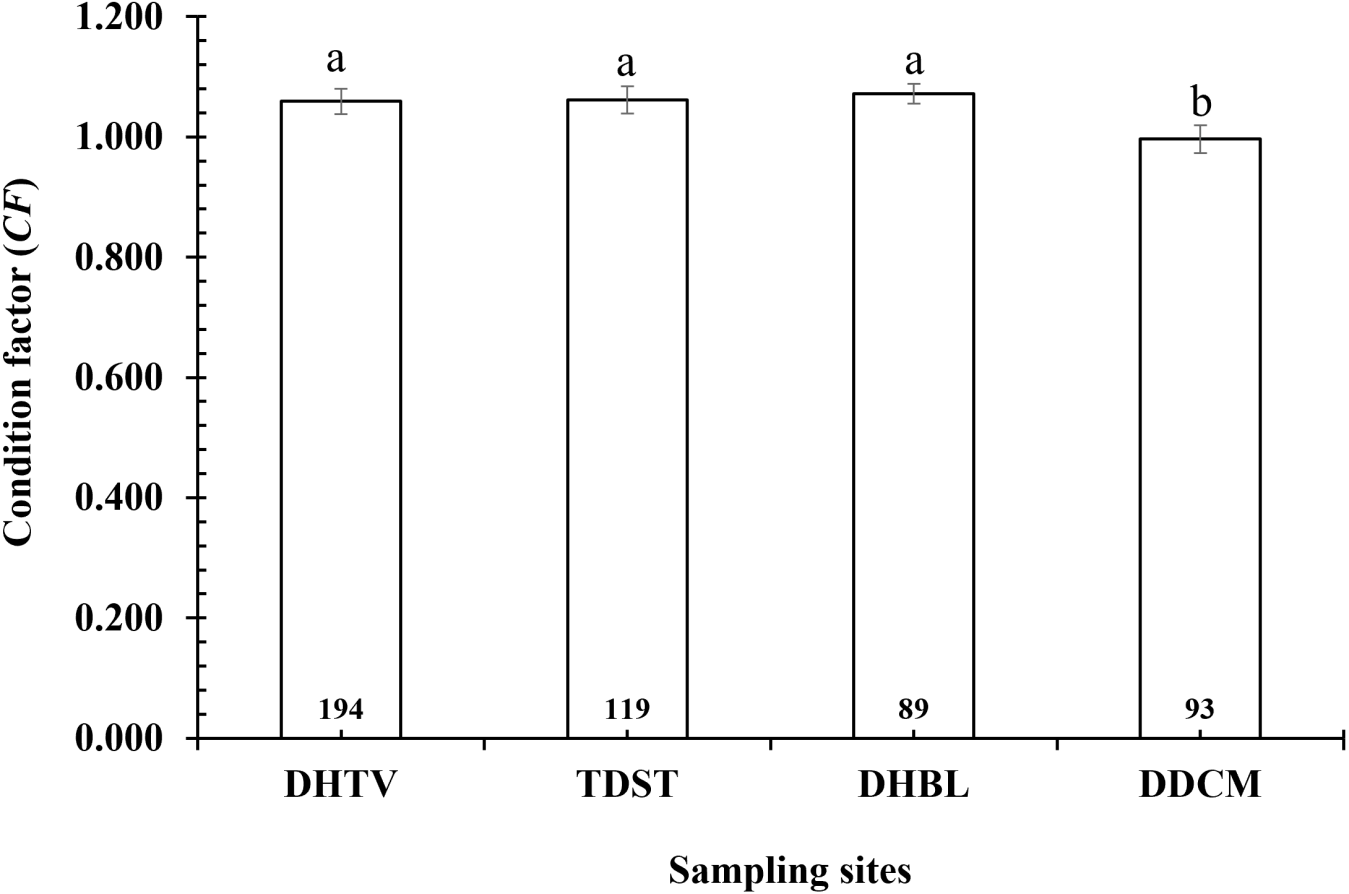
Variations of condition factor of *P. variabilis* by site. DHTV, Duyen Hai–TraVinh; TDST, Tran De–Soc Trang; DHBL, Dong Hai–Bac Lieu; DDCM, Dam Doi–Ca Mau; vertical bar is standard error of mean; a and b represent the significant difference; number in each column is number of samples.

**Figure 3:**
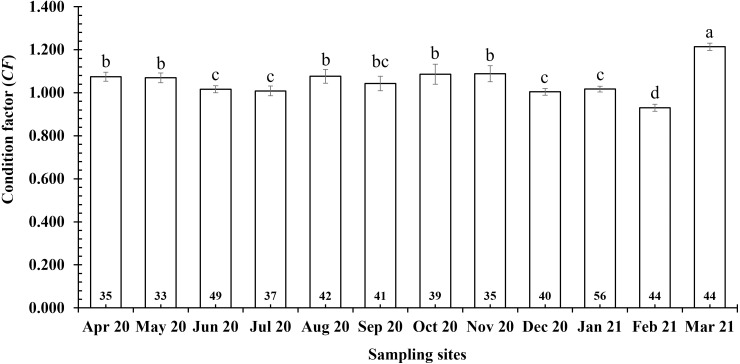
Variations of condition factor of *P. variabilis* by month. Letters a and b represent the significant difference; number in each column is number of samples.

**Figure 4:**
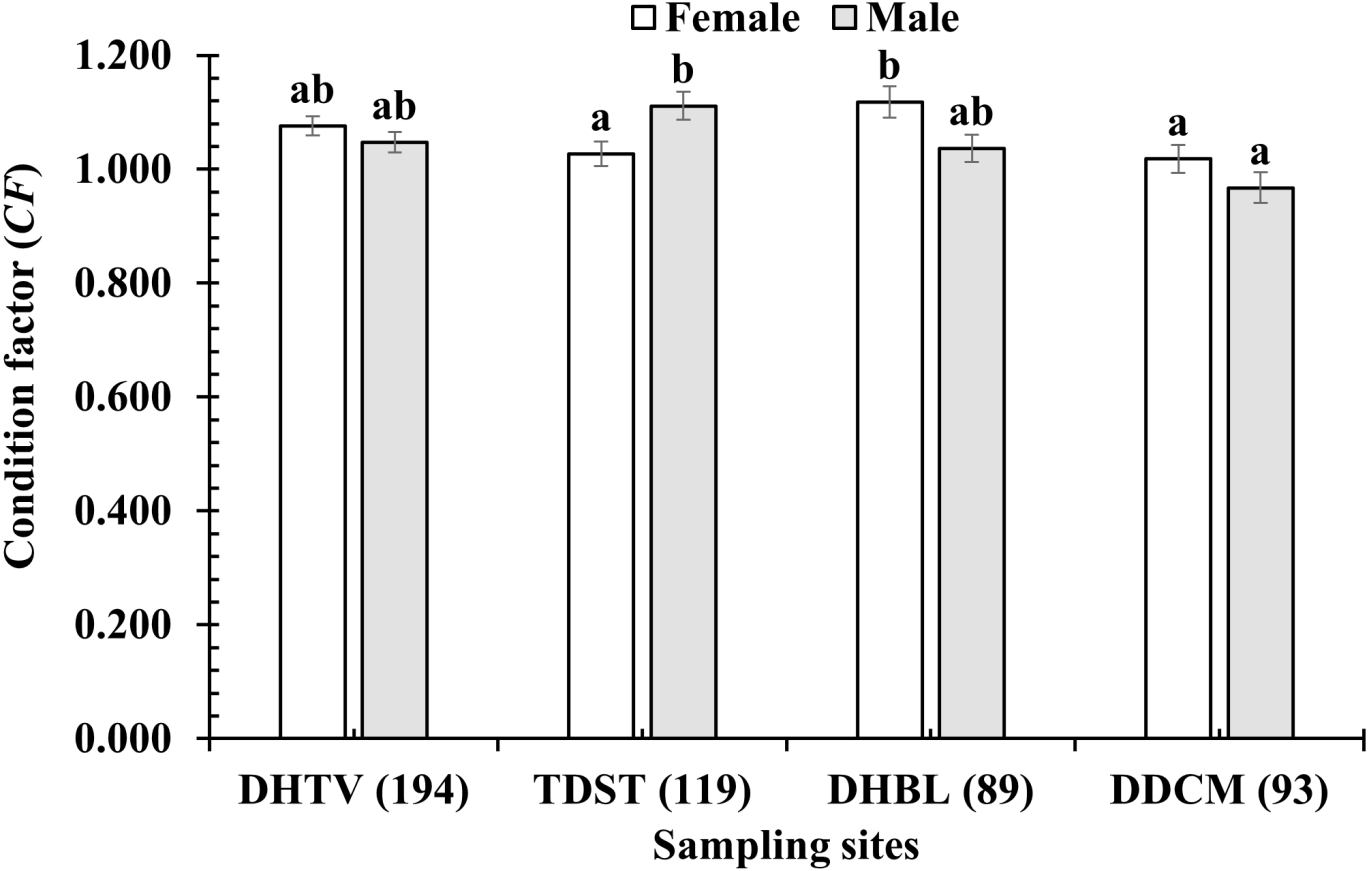
Variations of condition factor of *P. variabilis* by sex and site. DHTV, Duyen Hai–TraVinh; TDST, Tran De–Soc Trang; DHBL, Dong Hai–Bac Lieu; DDCM, Dam Doi–Ca Mau; vertical bar is standard error of mean; a and b represent the significant difference; number in parentheses is number of samples.

**Figure 5:**
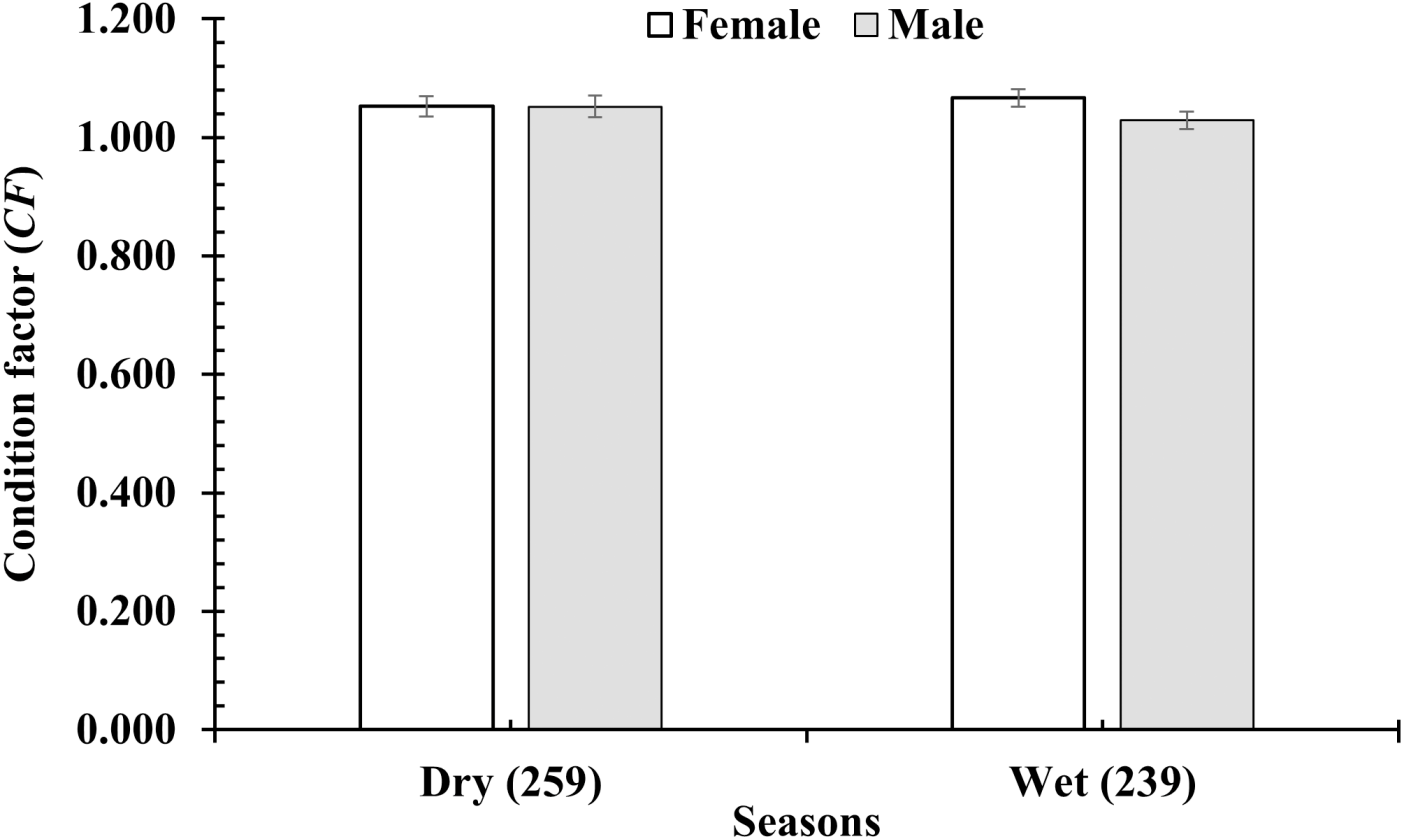
Variations of condition factor of *P. variabilis* by sex and season. Vertical bar is standard error of mean; number in parentheses is number of samples.

**Figure 6:**
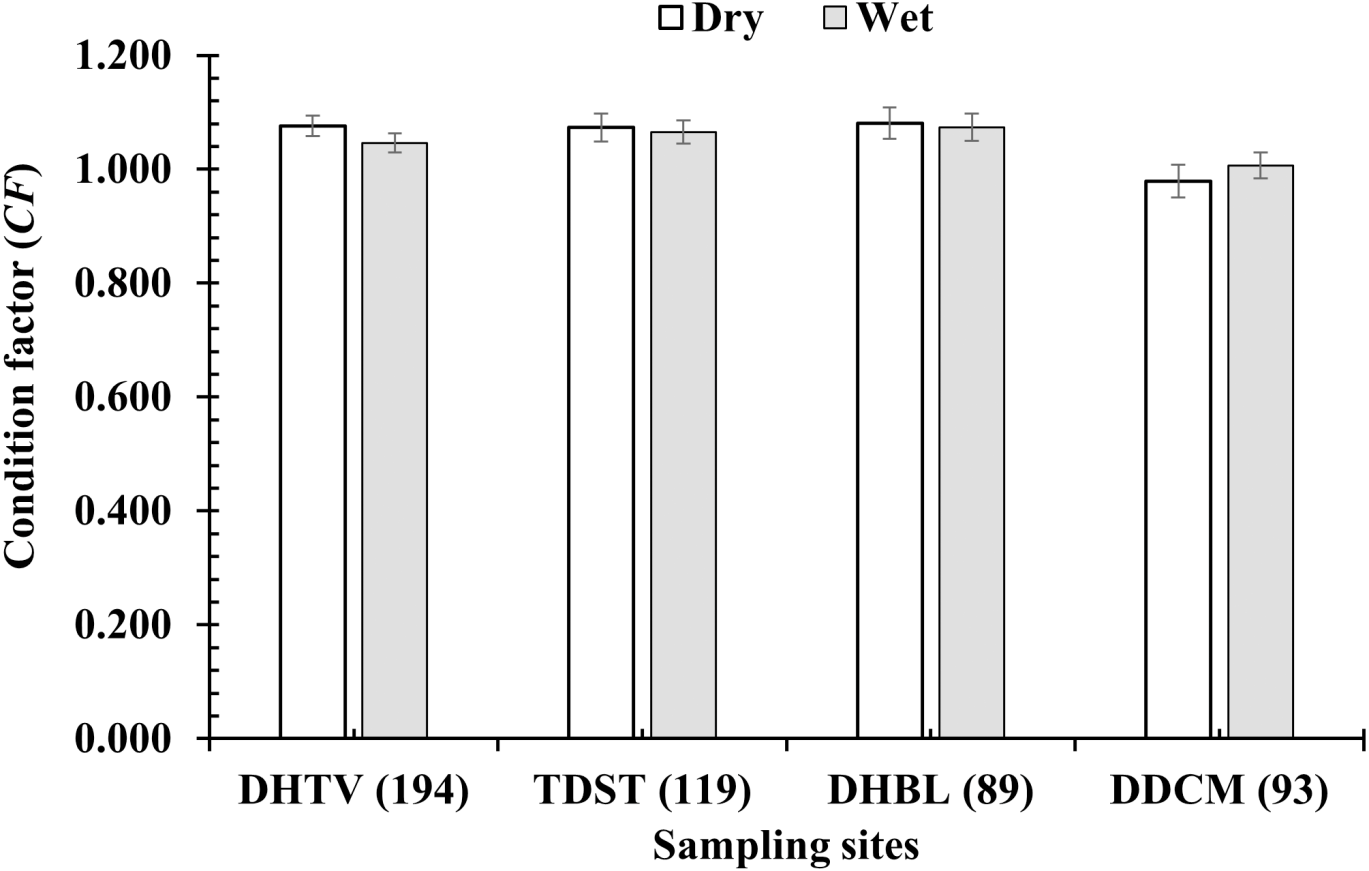
Variations of condition factor of *P. variabilis* by season and site. DHTV, Duyen Hai–TraVinh; TDST, Tran De–Soc Trang; DHBL, Dong Hai–Bac Lieu; DDCM: Dam Doi–Ca Mau; vertical bar is standard error of mean; number in parentheses is number of samples.

The *CF* of *P. variabilis* varied by site and month but not by sex, size or season. The *CF* of males (1.04 ± 0.01, *n* = 236) was not significantly different from that of females (1.06 ± 0.01, *n* = 259) (*t*-test, *t* = −1.05, *n* = 495, *df* = 493, *p* = 0.29, *CI*_95%_ = (−0.01)–(0.05)). The *CF* of the immature group (1.06 ± 0.03, *n* = 91) was similar to that of the mature group (1.05 ± 0.01, *n* = 404) (*t* =  − 0.47, *n* = 495, *df* = 493, *p* = 0.64, *CI*_95%_ = (−0.05)–(0.08)). The *CF* in the dry season (1.06 ± 0.01, *n* = 212) was not significantly higher than in the wet season (1.05 ± 0.01, *n* = 283) (*t* = −0.79, *n* = 495, *df* = 493, *p* = 0.43, *CI*_95%_ = (−0.02)–(0.04)). However, the *CF* in the DDCM was significantly lower in general than the remaining sites (One-way ANOVA, *n* = 495, *F*_2,3_ = 3.82, *p* = 0.01, Tukey Post Hoc comparison analysis) (Fig. 2). The *CF* all the sites in general also fluctuated from 0.93 ± 0.02 to 1.21 ± 0.02 (*n* = 495, *df* = 493, *F*_2,11_ = 7.69, *p* < 0.001) (Fig. 3) depending on the month. The *CF* of this species also varied by sex × site (General Linear Model, *n* =495, *n* = 495, *F*_2,3_ = 4.67, *p* = 0.003, Fig. 4), but not by sex × season (*F*_2,1_ = 1.30, *p* = 0.26, Fig. 5), season × site (*n* = 495, *F*_2,3_ = 0.41, *p* = 6.19, Fig. 6) or sex × season × site (*n* = 495, *df* = 1, *F*_2,3_ = 1.40, *p* = 0.24). Overall, the *CF* value of the mudskipper (1.05 ± 0.02, *n* = 495) was significantly higher than an ideal threshold value of 1 (*t* = 6.43, *n* = 495, *df* = 494, *p* < 0.001, *CI95%* = 0.04–0.07).

## Discussion

The positive relationship between weight and length was found in male, female, immature and mature *P. variabilis* due to the high values of the determination coefficients (*r*^2^) of the *LWRs*, suggesting that fish weight could be obtained from fish length regardless of the developmental stage of the fish. As high *r*^2^ values were shown in Tables 1 and 2 over a year long study, fish weight and length could be extrapolated using the data from such a large sample size and range. Similarly, a positive relationship between *TL* and *W* was found in *Periophthalmus* spp. living outside of the VMD (Chukwu & Deekae, 2011; Panicker, Katchi & Gore, 2013; Tran, Nguyen & Ha, 2021b). A positive *LWR* was also found in some other co-occurring gobies, *e.g.*, *Pseudapocryptes elongatus* (Tran, 2008), *Boleophthalmus boddarti* (Dinh, 2014b), *Glossogobius sparsipapillus* (Dinh, 2015), *P. serperaster* (Dinh et al., 2016), *Trypauchen vagina* (Dinh, 2016c), *Periophthalmodon schlosseri* (Dinh, 2016b), *Oxyeleotris urophthalmus* (Dinh, 2016a), *Butis butis* (Dinh, 2017b), *B. koilomatodon* (Lam & Dinh, 2021) and *G. giuris* (Dinh & Ly, 2014; Phan et al., 2021).

*P. variabilis* displayed positive allometry (*b* > 3) and the fish specimens collected were mostly adults, indicating that the fish population in this region was not overfished. By contrast, its congener, *e.g.*, *P. barbarus* in Nigeria, displayed negative allometry (*b* = 2.73), indicating that most *P. barbarus* were caught during the juvenile stage (King & Udo, 1998). Another study on the genus *Periophthalmus* in Indonesia showed that both *P. argentilineatus* and *P. gracilis* had negative allometry (*b* < 3) (Taniwel & Leiwakabessy, 2020). Negative allometry was also observed in some gobies living in the VMD, *e.g.*, *G. aureus* (*b* = 2.71) (Dinh, 2014a; Phan et al., 2021), *B. koilomatodon* (*b* = 2.66) (Lam & Dinh, 2021) and *G. sparsipapillus* (*b* = 2.68) (Truong et al., 2021). Meanwhile, other gobies living in the VMD exhibited isometric growth, *e.g.*, *B. boddarti* (Dinh, 2014b), *P. serperaster* (Dinh et al., 2016), *T. vagina* (Dinh, 2016c), *P. schlosseri* (Dinh, 2016b) and *G. giuris* (Dinh & Ly, 2014; Phan et al., 2021) with a *b* value of ≈3. Another congener of *P. variabilis*, *e.g.*, *P. modestus* found in the Red River Delta in northern Vietnam (VRD), showed positive allometry (*b* > 3) (Tran, Nguyen & Ha, 2021b). Positive allometry was also found in *P. chrysospilos* occurring in Malaysia (Abdullah & Zain, 2019) and *P. kalolo* and *P. malaccensis* in Indonesia (Taniwel & Leiwakabessy, 2020). Some other fish species living in the VMD, *e.g.*, *S. pleurostigma* displayed positive allometry as well (*b* > 3) (Dinh, 2017a). The similarities and differences in the growth pattern of *P. variabilis* and other gobies obtained from the *b* value of the *LWRs* suggest that fish growth type is species specific and regulated by the environment.

The growth pattern of *P. variabilis* did not change based on sex as both males and females showed isometry, suggesting that the different ovarian and testicular weights did not regulate fish growth type. A similar growth pattern in the two sexes was found in *P. barbarus* in Nigeria (King & Udo, 1998) but not in *P. modestus* in the VRD (Tran, Nguyen & Ha, 2021b). The growth pattern of *P. variabilis* was not impacted by size as evidenced by immature and mature groups both exhibiting isometry. This was also observed in *P. barbarus* in Nigeria (King & Udo, 1998) but not in *P. modestus* in the VRD (Tran, Nguyen & Ha, 2021b). Like its congener, *P. modestus* in the VRD (Tran, Nguyen & Ha, 2021b), the growth pattern of *P. variabilis* in the dry season was different from in the wet season, indicating that the difference in precipitation between these two seasons affected fish growth type. The various vegetation among the four sites could have an impact on *P. variabilis*’ growth patterns as well. The impact of environmental conditions on fish growth patterns was also found in *P. waltoni* in Nigeria (Sarafraz et al., 2012) and *P. modestus* in the VRD (Tran, Nguyen & Ha, 2021b). Like *P. variabilis*, some gobies living in and out of the VMD, *e.g.*, *P. barbarus* (King & Udo, 1998), *P. waltoni* (Sarafraz et al., 2012), *G. giuris* (Dinh & Ly, 2014; Phan et al., 2021), *B. boddarti* (Dinh, 2014b), *P. serperaster* (Dinh et al., 2016), *T. vagina* (Dinh, 2016c), *P. schlosseri* (Dinh, 2016b) and *P. modestus* (Tran, Nguyen & Ha, 2021b) showed variations in their growth patterns that fluctuated based on the month.

Like *B. koilomatodon* in the VMD (Lam & Dinh, 2021), the *CF* of *P. variabilis* was not affected by its developmental stage as its *CF* did not change with sex and size. Likewise, the *CF* of *P. variabilis’s* congener, *P. barbarus*, in Nigeria did not indicate changes in the *CF* based on sex differences (King & Udo, 1998; Chukwu & Deekae, 2011). By contrast, *P. modestus*, another congener in the VRD, showed a change in its *CF* due to sex as this value was high in females towards the end of gonadal maturation. Like *P. variabilis*, the *CF* of some gobies in the VMD, *e.g.*, *Parapocryptes serperaster* (Dinh et al., 2016), *P. schlosseri* (Dinh, 2016b), *T. vagina* (Dinh, 2016c) and *G. giuris* (Phan et al., 2021) did not vary with size. *P. variabilis* could adapt well to environmental conditions as its *CF* showed a similar pattern in the dry and wet seasons. In contrast, the dry season was preferable for *P. modestus* in the VRD as its *CF* value in the dry season was higher than it was in the wet season (Tran, Nguyen & Ha, 2021b). Like *P. variabilis*, a similar *CF* value was found between the dry and wet seasons in co-occurring gobiid species such as *P. elongatus* (Tran, 2008), *P. serperaster* (Dinh et al., 2016), *T. vagina* (Dinh, 2016c), *G. giuris* (Phan et al., 2021) and *B. koilomatodon* (Lam & Dinh, 2021). The difference in abiotic and biotic factors among sites (Dinh et al., 2021a) could be impacting the *CF* of *P. variabilis* as it showed a spatial variation in its *CF*. This same spatial variation in the CF was also found in the co-occurring goby species *B. koilomatodon* (Lam & Dinh, 2021) but was not found in the *P. modestus* species in the VRD (Tran, Nguyen & Ha, 2021b). Although *P. variabilis* showed spatiotemporal variations in its *CF*, overall its *CF* was higher than the threshold of 1 which could indicate that the study site had favourable environmental conditions. Congeners living outside of the VMD *e.g.*, *P. barbarus* (King & Udo, 1998), *P. chrysospilos* (Abdullah & Zain, 2019) and *P. modestus* (Tran, Nguyen & Ha, 2021b) were also well adapted to their habitats as evidenced by higher *CFs*. This was also observed in some other fish species in the VMD, *e.g.*, *P. elongatus* (Tran, 2008), *P. serperaster* (Dinh et al., 2016), *T. vagina* (Dinh, 2016c), *P. schlosseri* (Dinh, 2016b), *G. aureus* (Dinh, 2019), *P. chrysospilos* (Dinh et al., in press) and *G. giuris* (Phan et al., 2021).

## Conclusions

Due to high determination values fish weight can be estimated using a given fish length. As the slope value obtained from its *LWRs* was higher than the cubic value, *P. variabilis* displayed positive allometry. The growth pattern of this species was not affected by sexual and intraspecific factors since male, female, immature and mature fish displayed isometric growth as indicated by a *b* value ≈3. The *CF* was not affected by sex, size or season, but did vary by site and month. The *CF* of this species was higher than 1, showing it adapted well to the environment. These results can play a vital role in the assessment and management of the *P. variabilis* population in the VMD.

## Supplemental Information

10.7717/peerj.12798/supp-1 Supplemental Information 1 Raw data

## Additional Information and Declarations

### Data Availability

The following information was supplied regarding data availability: The raw data are available in the Supplementary File.
